# Incompatible effects of p53 and HDAC inhibition on p21 expression and cell cycle progression

**DOI:** 10.1038/cddis.2013.61

**Published:** 2013-03-07

**Authors:** M C C Sachweh, C J Drummond, M Higgins, J Campbell, S Laín

**Affiliations:** 1 Department of Microbiology, Tumor and Cell Biology, Karolinska Institutet, Stockholm 171 77, Sweden; 2 Centre for Oncology and Molecular Medicine, University of Dundee, Ninewells Hospital and Medical School, Dundee, Tayside DD1 9SY, UK

**Keywords:** trichostatin A, Nutlin-3, p53, p21, cancer therapy, iPS cell generation

## Abstract

Nutlin-3 selectively activates p53 by inhibiting the interaction of this tumor suppressor with its negative regulator murine double minute 2 (mdm2), while trichostatin A (TSA) is one of the most potent histone deacetylase (HDAC) inhibitors currently available. As both Nutlin-3 and TSA increase the levels of the cell cycle inhibitor p21(cip1/waf1) in cells, we investigated whether a combination of these compounds would further augment p21 levels. Contrary to expectations, we found that short-term exposure to Nutlin-3 and TSA in combination did not have an additive effect on p21 expression. Instead, we observed that activation of p53 prevented the ability of TSA to increase p21 levels. Furthermore, TSA inhibited Nutlin-3-induced expression of p53-dependent mRNAs including P21. This negative effect of TSA on Nutlin-3 was significantly less pronounced in the case of hdm2, another p53 downstream target. Aside from suggesting a model to explain these incompatible effects of Nutlin-3 and TSA, we discuss the implications of our findings in cancer therapy and cell reprogramming.

Small-molecule murine double minute 2 (mdm2)/p53 binding antagonists and histone deacetylase (HDAC) inhibitors are currently in clinical trials for cancer, and HDAC inhibitor suberoylanilide hydroxamic acid (SAHA; vorinostat; Zolinza) has been approved for the treatment of cutaneous T-cell lymphoma.^1^

Nutlin-3 is the most well-characterized mdm2/p53-binding antagonist. It binds to one of two sites in mdm2 occupied by the tumor suppressor p53 and protects the latter from mdm2-mediated degradation. As a consequence, Nutlin-3 selectively promotes cell cycle arrest and apoptosis in cells expressing wild-type p53.^2^ The activation of a variety of p53-dependent genes, including *CDKN1A* encoding p21(waf1/cip1) and *PIG3*, by Nutlin-3 is thought to mediate the majority of effects of this compound on cell proliferation and viability.^3^ However, Nutlin-3 is particularly effective at inducing the expression of *MDM2*, which is also a p53-dependent gene. The binding of Nutlin-3 to the mdm2 protein also protects mdm2 from degradation and stabilizes the structure of mdm2. Moreover, we have shown that following the removal of Nutlin-3 from cell cultures, p53 protein disappears within 30 min, while mdm2 levels remain constant for at least 3 h.^4^ Altogether, these results suggest that achieving a cytotoxic response following p53 reactivation may be difficult with mdm2/p53-binding antagonists.

Trichostatin A (TSA) is a natural compound and one of the most potent inhibitors of HDACs in both biochemical and cellular assays. TSA inhibits a wide range of HDACs, including all members of class I and II HDACs. Treatment with TSA for 24 h or longer has been reported to increase p53 acetylation and in this way protects p53 from degradation. In contrast, TSA has also been shown to reduce p53 expression by decreasing p53 promoter activity or even destabilization of P53 mRNA.^5, 6^ TSA and other HDAC inhibitors, including SAHA, do increase the expression of p21. However, unlike Nutlin-3, this activation of p21 expression is independent of the p53 status in cells.^7, 8, 9, 10, 11, 12^

With the purpose of enhancing the activity of p53, we have combined Nutlin-3 with a number of p53-modulating compounds. Here we present the data obtained using a combination of Nutlin-3 and TSA in different cell lines.

## Results

### TSA reduces the induction of p53-dependent genes by Nutlin-3

**Figure 1:**
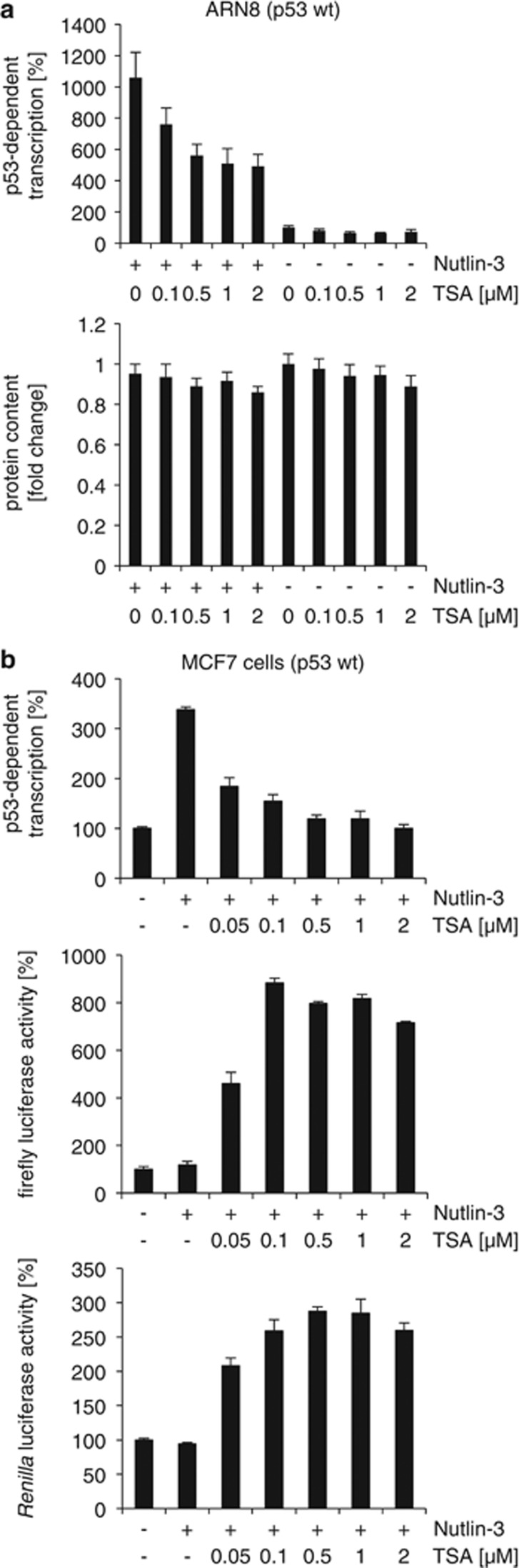
TSA reduces p53-dependent transcription in Nutlin-3-treated cells. (**a**) ARN8 melanoma cells containing wild-type p53 and the p53-dependent ΔFos-RGC-LacZ reporter plasmid were treated with TSA and/or Nutlin-3 (2 *μ*M) for 16 h as indicated. *β*-Gal p53 reporter activity (top panel) and protein content (bottom panel) were measured. (**b**) MCF7 cells transiently transfected with the p53-dependent ΔFos-RGC-LacZ reporter plasmid together with a control plasmid expressing firefly or *Renilla* luciferase under the control of the SV40 promoter. At 24 h post-transfection, cells were treated with TSA and/or Nutlin-3 (5 *μ*M) for 16 h as indicated and reporter activities measured. Error bars represent standard deviation

As a first approach to analyze whether Nutlin-3 and TSA cooperate in promoting p53-dependent transactivation, we tested their effects on an artificial p53-responsive reporter. In particular, we used ARN8 and MCF7 cells harboring wild-type p53 and a construct encoding the *LACZ* gene under the control of a p53-responsive promoter. As expected, Nutlin-3 on its own increased p53-dependent transcription, whereas TSA had no positive effect (Figure 1). Unexpectedly, when Nutlin-3 and TSA were combined, p53 reporter activity was reduced, suggesting that TSA has a negative effect on Nutlin-3-induced p53 transcription factor function.

**Figure 2:**
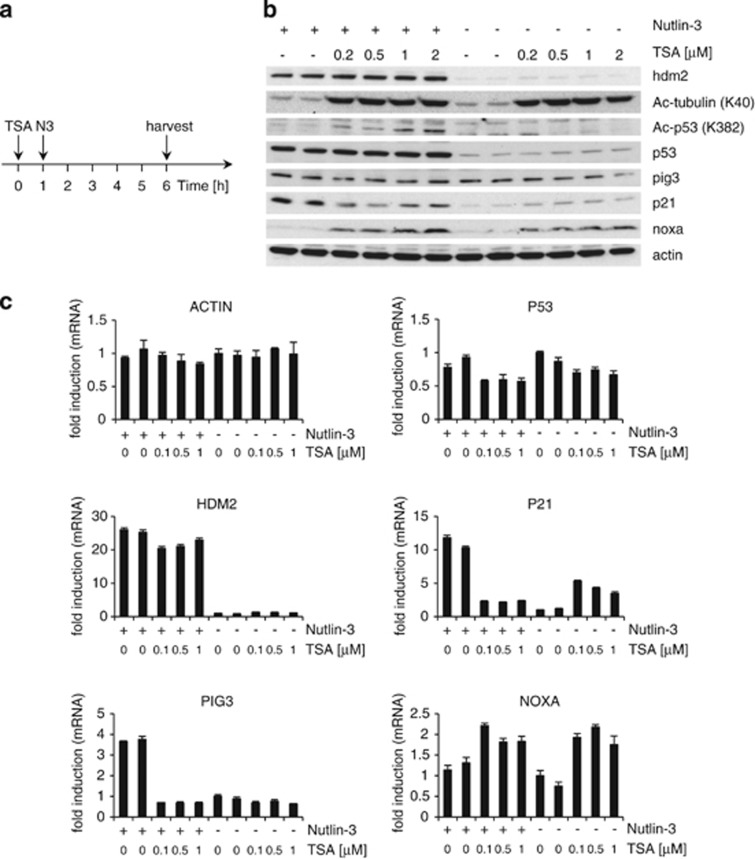
Effects of TSA pre-treatment on the expression of Nutlin-3-responsive genes in MCF7 tumor cells. (**a**) Cells were mock-treated (ethanol (EtOH)) or treated with TSA at various concentrations for 6 h; 1 h after TSA treatment, Nutlin-3 (5 *μ*M) or vehicle control (dimethyl sulfoxide (DMSO) or EtOH) was added. Target gene expression was determined by (**b**) western blotting and (**c**) quantitative RT-PCR. (**b**) Acetylated *α*-tubulin (K40) was used as control for TSA's activity and actin as a loading control. (**c**) Error bars represent standard deviation. Graphs shown are representative of three independent experiments; the other two sets of data are shown in Supplementary Figure S1A

We then analyzed the effects of Nutlin-3, TSA and their combination on mRNA expressed from p53 downstream target genes in MCF-7 cells (Figures 2a and c and Supplementary Figure S1A). Nutlin-3 increased the expression of p53-dependent mRNAs following 5 h of exposure. TSA on its own induced an increase in P21 mRNAs as well as a small but reproducible rise in HDM2 (human MDM2) mRNA. In contrast, pre-treatment of cells with TSA clearly reduced P21 and PIG3 mRNA levels in Nutlin-3-treated cells. This negative effect of TSA on Nutlin-3 activation of HDM2 mRNA expression was less evident. Interestingly, the expression of the proapoptotic gene *NOXA*, which was identified as being p53-dependent,^13^ was increased by TSA regardless of the presence of Nutlin-3. This suggests that TSA does not reduce mRNA levels of all genes. Supporting this, TSA also had a positive effect on luciferase reporter expression (Figure 1b).

In MCF7 cells, the induction of p21 protein by Nutlin-3 was reduced when cells were pre-treated with TSA, although to a lesser extent than P21 mRNA levels (Figure 2b and Supplementary Figure S2A). The induction of pig3 at the protein level by short exposure to Nutlin-3 was very weak. Even so, TSA caused a slight reduction in Nutlin-3-induced pig3 protein levels. Hdm2 protein levels were highly induced by Nutlin-3, and this induction was insensitive to TSA pre-treatment. Noxa protein levels were clearly increased by TSA in the presence or absence of Nutlin-3.

**Figure 3:**
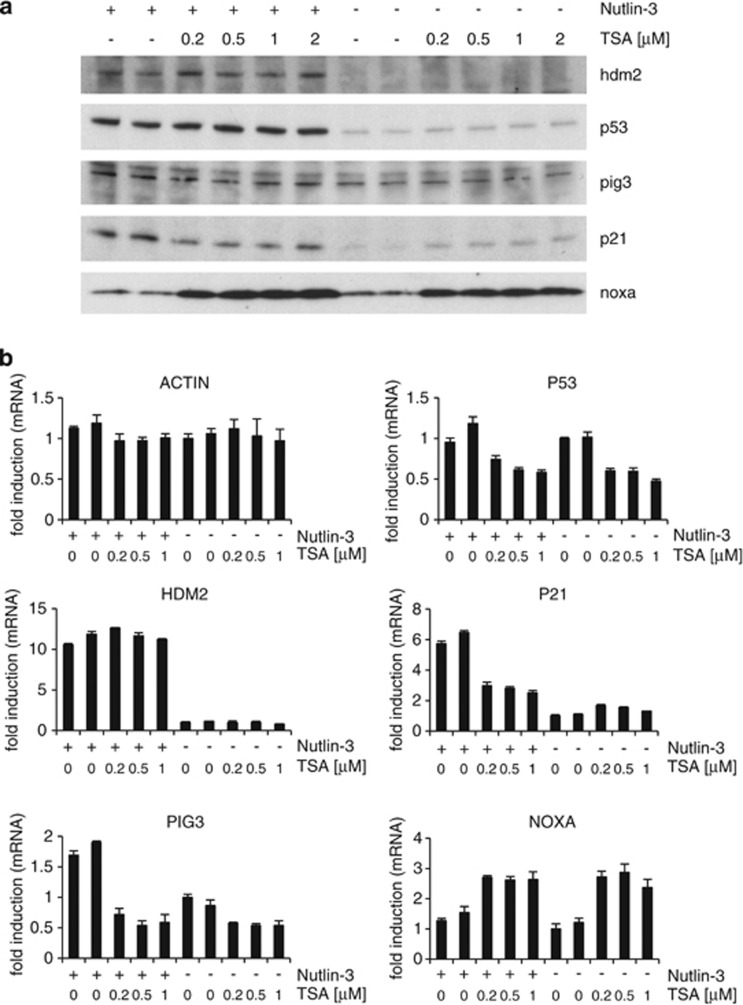
Effects of TSA pre-treatment on the expression of Nutlin-3-responsive genes in HNDFs. Samples were treated and analyzed as in Figure 2. Error bars represent standard deviation. Graphs shown are representative of three independent experiments; the other two sets of data are shown in Supplementary Figure S1B

Similar results were obtained in human normal dermal fibroblasts (HNDFs) (Figure 3 and Supplementary Figures S1B and S2B), although TSA on its own did not raise HDM2 mRNA levels and there was no negative effect of TSA on Nutlin-3 activation of HDM2 mRNA expression.

### Short-term TSA treatment leads to a small reduction in P53 mRNA levels but not in p53 protein levels

In MCF7 cells, P53 mRNA levels were insensitive to Nutlin-3 and slightly reduced by TSA on its own as well as in the presence of Nutlin-3 (Figure 2c). This is in line with reports showing that TSA reduces P53 mRNA expression and/or the stability of P53 mRNA.^5, 6^ However, p53 protein levels were not reduced in response to short-term treatment with TSA in any of the conditions tested and regardless of the anti-p53 antibody used (Supplementary Figure S3). In addition, we did not detect changes in p53's subcellular localization induced by TSA (Supplementary Figures S3 and S4). Instead, acetylation of p53 at residue K382, which is associated with p53 stabilization, was increased by TSA in combination with Nutlin-3 (Figure 2b). Similar results were obtained in HNDF cells (Figure 3).

### TSA weakens Nutlin-3-induced G1 arrest

Nutlin-3 treatment is known to cause cell cycle arrest in cells expressing wild-type p53 and this effect is highly dependent on its ability to increase p21 expression.^14^

**Figure 4:**
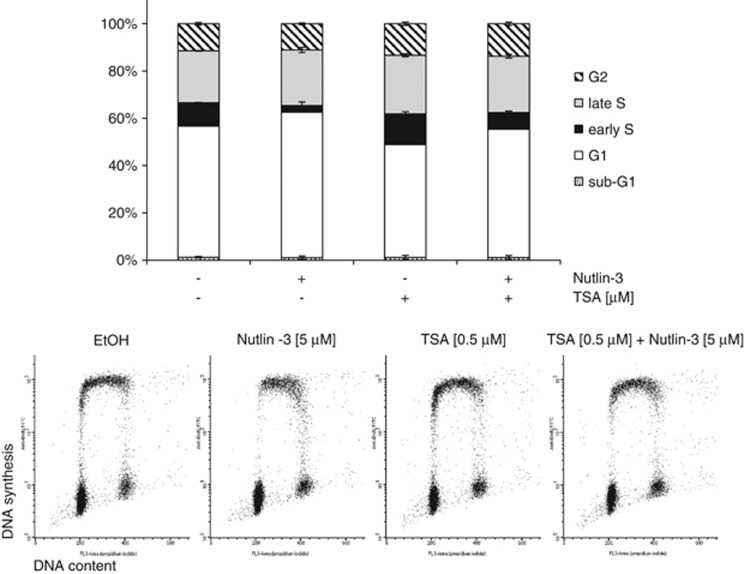
TSA weakens Nutlin-3-induced G1 arrest. MCF7 cells were treated as in Figure 2a. Samples were subjected to two-dimensional flow cytometric analysis following staining with BrdU and PI. Error bars represent standard deviation (*n*=4)

As shown in Figure 4 and Supplementary Figure S5, in MCF-7 cells, even a short exposure to Nutlin-3 led to an increase in the proportion of G1-phase cells (*P*<0.005) and a reduction in early S-phase cells (*P*<0.0001). Interestingly, if cells were pre-treated with TSA, this Nutlin-3-induced reduction in early S-phase cells was less apparent (*P*<0.0001). Note that TSA increased the proportion of early S-phase cells also in the absence of Nutlin-3 (*P*<0.005). These observations show that in the short term, TSA can prevent Nutlin-3-induced G1 arrest. This is in agreement with previous reports of the positive effect of TSA on the S-phase entry.^15, 16^

### p53 reduces TSA's ability to activate p21 expression

**Figure 5:**
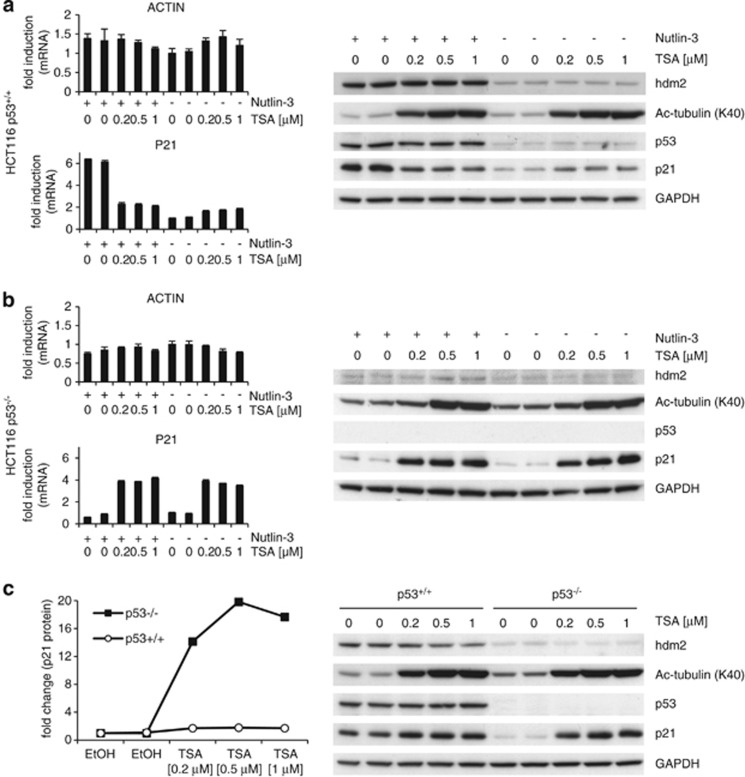
p53 reduces TSA's ability to activate p21 expression. (**a**) HCT116 p53^+/+^ and (**b**) HCT116 p53^−/−^ cells were treated and analyzed as in Figure 2. (**b**) Acetylated *α*-tubulin (K40) was used as control for TSA's activity and GAPDH as a loading control. (**c**) Error bars represent standard deviation. Graphs shown are representative of three independent experiments; the other two sets of data are shown in Supplementary Figures S1C and S1D, respectively. (**c**) HCT116 p53^+/+^ and HCT116 p53^−/−^ cells were treated with vehicle (EtOH) or various concentrations of TSA for 6 h. Protein expression of p53 and p53 target genes were analyzed by western blotting (right panel); p21 protein levels were analyzed densitometrically (left panel)

It is well established that TSA increases p21 expression independently of p53.^7, 8, 9, 10^ Indeed, a short treatment with TSA markedly increased P21 mRNA and protein levels in MDA-MB-468 cells carrying mutant p53 (Supplementary Figure S6). Our previous experiments have shown that TSA can activate p21 expression in cells retaining wild-type p53 (Figures 2 and 3 and Supplementary Figures S1 and S2). However, at least at the protein level, we noticed that the effect of TSA in these cells was much weaker than in MDA-MB-468 cells. This suggests that wild-type p53 prevents p21 induction by TSA. To confirm this, we used HCT116 p53^+/+^ and HCT116 p53^−/−^ isogenic cell lines. TSA led to a much stronger increase in P21 mRNA and protein levels in HCT116 p53^−/−^ cells than in their wild-type p53 counterparts (Figure 5 and Supplementary Figures S1C, S1D and S2C, S2D).

**Figure 6:**
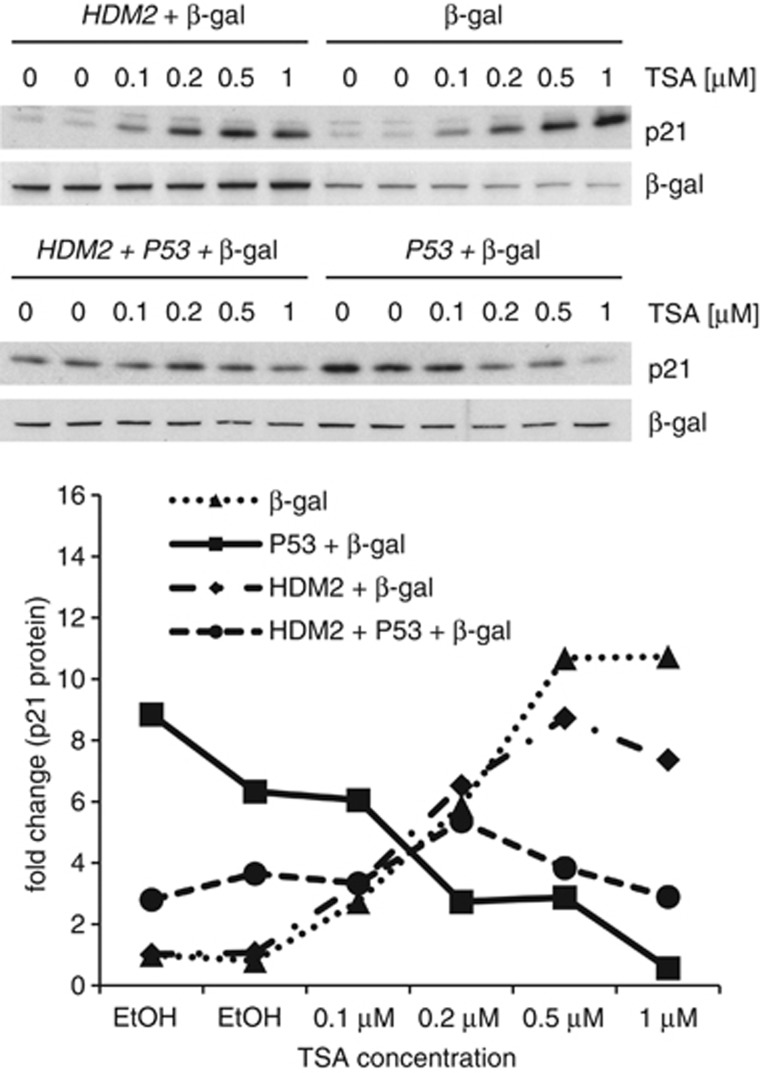
p53 inhibits the induction of p21 by TSA. H1299 (p53-null) cells were transfected with expression vectors encoding hdm2 and/or human p53. At 24 h post-transfection, cells were treated with TSA for 6 h. All samples were transfected with a *β*-gal expression construct to assess sample loading and transfection efficiency. Protein levels were assessed by western blotting (upper two panels) and subsequent densitometric analysis (lower panel)

In a series of transfection assays, we observed that in the presence of transfected p53, TSA had a negative effect on p21 expression (Figure 6). Furthermore and as expected, this negative effect was weakened when hdm2 was coexpressed. Altogether, these results show that active p53 has a negative effect on TSA's ability to induce p21.

**Figure 7:**
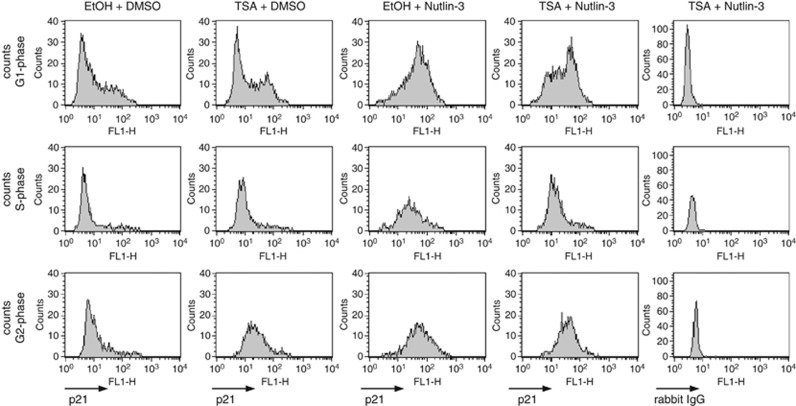
Nutlin-3 increases p21 levels during all phases of the cell cycle, whereas TSA increases p21 in cells during the G1 and G2 phase. MCF7 cells were subjected to short-term treatment with TSA (0.5 *μ*M) and/or Nutlin-3 (5 *μ*M) as indicated in Figure 2a. Fixed cells were incubated with an antibody against p21 (first four columns) or rabbit IgG (fifth column), respectively, stained with PI and subjected to flow cytometric analysis

We also determined at what phase in the cell cycle p21 is induced by TSA or Nutlin-3. As shown in Figure 7, Nutlin-3 increased p21 protein levels in all phases of the cell cycle, whereas TSA increased p21 only in G1 and G2 phases. Therefore, it is unlikely that the reciprocal negative effects of TSA and Nutlin-3 on p21 expression are due to cell cycle arrest at a stage where p21 cannot be induced by one of these compounds.

### p53 activation by Nutlin-3 reduces TSA's ability to cause G2/M arrest and endoreduplication in tumor cells

**Figure 8:**
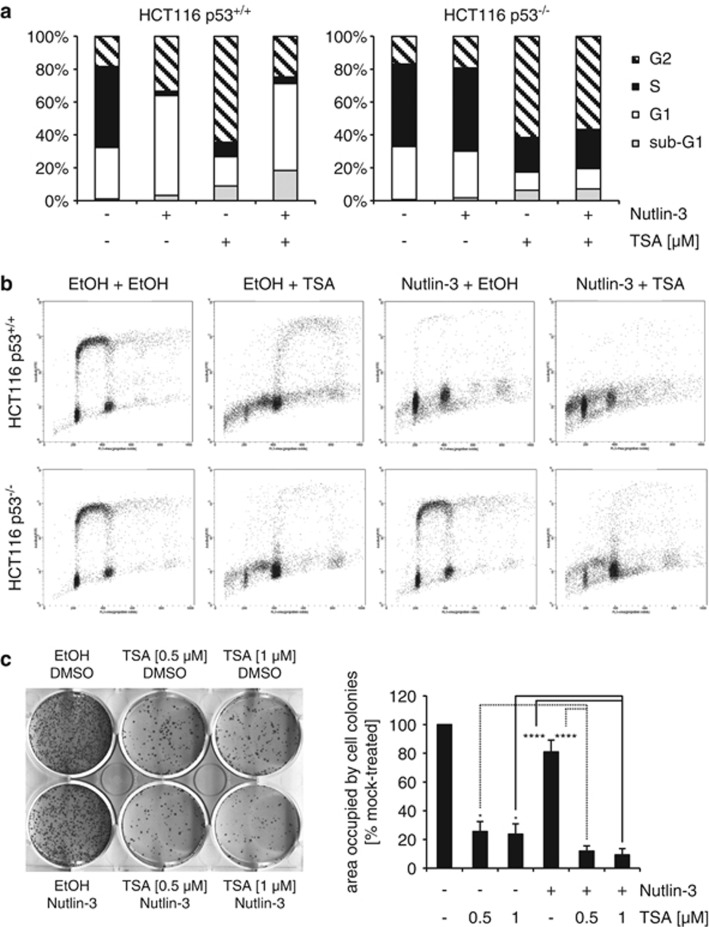
Nutlin-3 reduces TSA's ability to cause G2/M arrest and endoreduplication in tumor cells but does not protect tumor cells from TSA-mediated cytotoxicity. (**a** and **b**) HCT116 p53^+/+^ or HCT116 p53^−/−^ cells were mock-treated (EtOH) or treated with Nutlin-3 (5 *μ*M) for 24 h after which TSA (0.5 *μ*M) was added for 30 h. Cell cycle distribution was determined by two-dimensional flow cytometry for BrdU and PI. (**c**) HCT116 p53^+/+^ cells were treated for 24 h with TSA and/or Nutlin-3 (5 *μ*M), the latter being added 1 h after TSA treatment. Afterwards, cells were washed with growth medium and allowed to grow for 9 days. Fixed cells were stained with Giemsa solution. The histogram shows plate area occupied by cell colonies as quantified in ImageJ; plate area occupied by mock-treated cells was set at 100%. Error bars represent standard deviation of three independent experiments. *P<0.05; ****P<0.001 (unpaired two-tailed Student's *t*-test; *n*=3)

Long-term treatment with TSA can lead to an arrest of cells in the G2 or G2/M phase of the cell cycle.^17, 18^ In agreement with this, after longer exposure to TSA (30 h), the great majority of cells in the culture had a 4*N* DNA content (Figure 8a). TSA-induced G2/M arrest and endoreduplication occurred in HCT116 cells regardless of whether they contain wild-type p53 or not. However, both the G2/M arrest and the endoreduplication events were reduced in cells pre-treated with Nutlin-3. As expected, this protective effect of Nutlin-3 was restricted to cells that express wild-type p53 (Figures 8a and b).

Nevertheless, as shown in a clonogenic assay (Figure 8c), cotreatment of HCT116 p53^+/+^ cells with Nutlin-3 and TSA is more toxic than either treatment on its own. Therefore, in the long term, Nutlin-3 does not substantially protect tumor cells from toxicity induced by TSA. In contrast, Nutlin-3 is known to protect HCT116 p53^+/+^ cells from cytotoxic agents (i.e. S- and M-phase inhibitors) very effectively.^19, 20, 21, 22, 23^

## Discussion

We sought to identify small molecules that could enhance the activity of p53 by combining the mdm2/p53-binding antagonist Nutlin-3 with small molecules known to modulate p53 function. One of the compounds tested was TSA, which is known to affect p53 expression as well as its stability. Contrary to our initial expectations from experiments performed by long-term treatment of cells with TSA in other labs, our results clearly showed that short-term treatment with this HDAC inhibitor reduced the expression of p53 transcription factor function. In addition, activated p53 reduced the ability of TSA to induce p21 expression. In the next paragraphs, we propose a series of explanations for the observed effects of the short-term cotreatment with TSA and Nutlin-3 on the expression of hdm2, p21 and pig3.

### TSA has a minor effect on hdm2 expression

In the absence of Nutlin-3, TSA did not have a substantial negative effect on HDM2 mRNA levels, whereas in the presence of Nutlin-3, TSA reduced HDM2 mRNA levels to a small extent in some cell lines (i.e. MCF7 and HCT116 p53^+/+^, see Figure 2 and Supplementary Figure S7). This small reduction in HDM2 mRNA levels could be explained by the small TSA-induced decrease in P53 mRNA observed in these cells. In HNDFs cotreated with TSA and Nutlin-3, there was no reduction in HDM2 mRNA levels in response to TSA, even when P53 mRNA levels were reduced by TSA (Figure 3). The lack of effect on HDM2 mRNA levels in HNDF cells may be related to the ability of TSA to induce the expression of p14^ARF^.^24^ p14^ARF^ is a tumor suppressor that activates p53 and whose expression is impaired in most tumor cell lines retaining wild-type p53 including MCF7 and HCT116 cells.^25^ Overall, the effect of TSA on HDM2 mRNA was either very low or imperceptible and TSA-induced variations on endogenous hdm2 protein levels were negligible in comparison with the effects of TSA on p21 and pig3, as discussed below.

### p53 weakens the positive effect of TSA on p21

In the case of P21 mRNA, TSA on its own clearly increased its levels in all cell lines tested (Figures 2, 3 and 5 and Supplementary Figures S1 and S6). Interestingly, the increase in P21 mRNA and protein levels by TSA was significantly more pronounced in HCT116 p53^−/−^ cells than in HCT116 p53^+/+^ cells (Figure 5c and Supplementary Figure S2). It has been described previously that TSA is able to induce p21 expression by increasing the levels of acetylated p300.^11^ p300 is an acetyltransferase that becomes activated upon acetylation and is involved in the activation of transcription of a large number of genes (reviewed in Chen and Li^26^). It has further been shown that activation of p300 by TSA leads to cooperation between this protein and the transcription factors Sp1 and Sp3^11^ through another transcription factor, that is, ZBP-89 (BFCOL1/BERF-1/ZNF-148). Eventually, this complex activates *CDKN1A* (p21) transcription in the absence of p53.^10, 12^

Our data further indicate that TSA fails to increase P21 mRNA and protein levels in cells expressing wild-type p53 and treated with Nutlin-3 (Figures 2, 3 and 5 and Supplementary Figure S1). This suggests that active p53 prevents the ability of TSA to increase p21 expression. Another explanation may be that TSA's increasing effect on *CDKN1A* (p21) transcription is linked to its ability to reduce c-myc levels,^27, 28^ as c-myc can reduce p21 levels.^29, 30, 31, 32^ Because p53, like TSA, represses the *C-MYC* promoter,^33, 34^ further inhibition of c-myc expression by TSA could have no consequence.

### TSA inhibits the effect of Nutlin-3 on p21

The previously reported reduction of c-myc levels by TSA^27, 28^ may also explain why in the presence of TSA, Nutlin-3 fails to increase p21 levels further. Yet, this explanation is insufficient to understand why TSA markedly reduces P21 and PIG3 mRNA levels in the presence of Nutlin-3. As suggested above, TSA caused a small reduction in HDM2 mRNA levels in MCF7 and HCT116 cells cotreated with Nutlin-3 that could be explained by a small reduction in *de novo* synthesis of p53. However, although this small decrease in p53 synthesis by TSA may be the underlying cause, it is not sufficient to understand our observations on P21 and PIG3 mRNA levels, for which the negative effects of TSA in Nutlin-3 cotreated cells were much more pronounced. In a more extreme situation, that of HNDFs, HDM2 mRNA levels did not decrease at all, whereas P21 and PIG3 mRNAs were reduced substantially (Figure 3).

This selectivity could be due to a lower sensitivity of the *HDM2* promoter to reductions in newly synthesized p53. To understand why the *CDKN1A* (p21) promoter would be more sensitive to a small reductions in p53 than the *HDM2* promoter in nutlin-3-treated cells, we propose a model based on the two following reports: First, it has been shown that *CDKN1A* and *PIG3* promoters are bound by p53 and hdm2 and that the binding of hdm2 is associated with a reduction in the promoters' activity. In contrast, the *HDM2* promoter is not bound by hdm2.^32^ Second, in the presence of Nutlin-3, HDM2 mRNA and protein levels are particularly high relative to other p53 downstream products.^4^ With this evidence, it is not unreasonable to suggest that the sharp hdm2 induction caused by Nutlin-3 may contribute in enhancing the TSA-induced reduction in the expression of p53-dependent genes that are inhibited by hdm2, such as *CDKN1A*, but not *HDM2*. This interpretation could be extended to rationalize the negative effect of TSA on Nutlin-3-induced PIG3 mRNA. However, in this case, it must be kept in mind that TSA may have a negative effect on PIG3 mRNA expression regardless of the presence of p53. Note that a reduction in PIG3 mRNA levels by TSA also occurs in HCT116 cells where p53 is depleted (Supplementary Figure S7).

### TSA is the only small-molecule p53 inhibitor with known targets

Pifithrins-*α* and -*μ* (reviewed in Gudkov and Komarova^35^) are two examples of small molecules that reduce p53 activity. However, the targets for pifithrins are still unknown. Instead, the mode of action of TSA is well-characterized. Even so, because TSA targets a wide range of HDACs involved in regulating a myriad of factors, understanding exactly how TSA treatment leads to a reduction in the expression of p53-dependent genes in cells cotreated with Nutlin-3 is a difficult task. Nevertheless, our work makes TSA the only compound inactivating p53 for which there is an established mode of action.

### Implications for cancer therapy

From a cancer therapy perspective, our observations on the incompatible effects of TSA and Nutlin-3 on p21 expression may be of relevance when evaluating regimens where HDAC inhibitors and mdm2/p53-binding antagonists are combined. On the one hand, the results presented here could suggest that treatment of p53 wild-type tumor cells with an HDAC inhibitor could weaken the antitumor effect of Nutlin-3 and *vice versa*. On the other hand, it could be argued that by diminishing P21 mRNA levels, TSA could reduce Nutlin-3's cytostatic effect and increase the proportion of cells undergoing apoptosis. However, this interpretation is debatable, as it has been shown that p21 does not necessarily protect cancer cells from apoptosis induced by Nutlin-3.^14^ In any case, our results show that even if Nutlin-3 and TSA may weaken each others' effects in the short-term, long-term cotreatment of HCT116 p53^+/+^ cells with Nutlin-3 and TSA is still more toxic than either treatment on its own (Figure 8c). This is very different from experiments where Nutlin-3 treatment clearly protects HCT116 p53^+/+^ cells from the S-phase and mitotic poisons.^19, 20, 21, 22, 23^

### Relevance for the generation of induced pluripotent stem cells

Another interesting feature of TSA is that it increases efficiency in protocols to obtain induced pluripotent stem cells (iPS cells).^36^ Most likely, this is due to the effects of TSA on histone acetylation and removal of epigenetic inhibition of stem cell genes and pluripotency-associated genes.^37^ Our results suggest that part of TSA's positive effect in iPS cell protocols could be due to its ability to reduce the activity of p53. This is of relevance as it has been shown by four independent labs that deletion of p53 substantially increases efficiency in iPS cell protocols (reviewed in Okita and Yamanaka^38^). In this regard, it is worth noting that the expression of pluripotency genes *OCT4*, *SOX2*, *C-MYC* and *KLF4* used to obtain iPS cells induces p53 and p21 levels.^39^ However, and even if treatment with TSA (unlike genetic deletion of *P53*) is not permanent, it must be borne in mind that the ability of TSA to reduce p53 transcription factor function could also contribute to genomic abnormalities in iPS cell cultures.

## Materials and Methods

### Cell culture

ARN8 cells,^40^ MCF7 cells (ATCC, Teddington, UK) and HNDFs (no. C-12300; PromoCell, Heidelberg, Germany) were cultured in DMEM supplemented with 10% FBS and 1% penicillin/streptomycin (P/S). H1299 cells (ATCC) were grown in RPMI-1640 medium supplemented with 10% FBS and 1% P/S. HCT116 p53^+/+^ and HCT116 p53^−/−^cells (a kind gift from B Vogelstein^41^) were cultured in McCoy's 5A medium supplemented with 10% FBS, 1% P/S and 3 mM ℒ-glutamine.

### Reagents and antibodies

Nutlin-3 (no. N6287), TSA (no. T8552), formaldehyde (no. F1635), ribonuclease A (RNase A; no. R4642), Giemsa solution (no. 32884) and bovine serum albumin (BSA; no. 9647) were obtained from Sigma-Aldrich (Heidelberg, Germany). Chlorophenol red-*β*-𝒟-galactopyranoside (CPRG; no. 884308) and FuGene 6 (no. 12454000) were purchased from Roche (Mannheim, Germany). Propidium iodide (PI; no. P3566) was obtained from Invitrogen (Eugene, OR, USA).

Antibodies used for western blotting were as follows: anti-Ac-p53 (K382) (no. 61420; BioLegend, London, UK), anti-actin (no. CP01; Calbiochem, Darmstadt, Germany), anti-*β*-galactosidase (*β*-gal) (no. OB02; Calbiochem), anti-glyceraldehyde 3-phosphate dehydrogenase (GAPDH; no. 10R-G109a; Fitzgerald, Acton, MA, USA), anti-hdm2 (4B2; a kind gift from David P Lane),^42^ anti-noxa (no. ab13654; Abcam, Cambridge, UK), anti-p21 (118; a kind gift from David P Lane),^43^ anti-p53 (DO-1; a kind gift from David P Lane)^44^ and anti-pig3 (no. sc-2020; Santa Cruz Biotechnology, Heidelberg, Germany). Secondary antibodies used for western blotting were horseradish peroxidase (HRP)-conjugated rabbit anti-mouse (no. P0161; Dako, Glostrup, Denmark) and HRP-conjugated swine anti-rabbit (no. P0217; Dako). Antibodies used for flow cytometry were anti-5-bromo-2′-deoxyuridine (BrdU; no. 347580; Becton Dickinson, Franklin Lakes, NJ, USA), fluorescein isothiocyanate-conjugated sheep anti-mouse (no. F3008; Sigma-Aldrich), Alexa Fluor 488-conjugated anti-p21 (no. 5487; Cell Signaling, Danvers, MA, USA) and Alexa Fluor 488-conjugated rabbit IgG (no. 4340; Cell Signaling).

### p53-dependent transcription measurements

ARN8 cells expressing (*β*-gal) under the control of a p53-dependent promoter (RGCΔFos-LacZ) were assayed as described previously.^45^ MCF7 cells were transiently transfected using FuGene 6 with both a plasmid containing the RGCΔFos-LacZ construct^46^ and a plasmid encoding either Renilla or firefly luciferase; the latter two are driven by the SV40 promoter. After incubation with compounds for 24 h, cells were lysed and *β*-gal activity detected using CPRG as a substrate.^45^ To measure luciferase activity, MCF7 cells were lysed with 1 × Reporter Lysis Buffer (no. E397A; Promega, Madison, WI, USA), equal volumes of substrate from the Dual-Luciferase Reporter Assay System (no. E1960; Promega) added and luminescence measured on a Microplate Luminometer LB 96V (EG & G Berthold, Bad Wildbad, Germany). RNA preparation and quantitative reverse transcription-polymerase chain reaction (quantitative RT-PCR) analysis were performed as described previously.^4^

### Western blotting

Samples were prepared as described previously^4^ and run on 4–12% precast gels (nos. NP0321 and NP0322; Invitrogen) according to the manufacturer's instructions. Proteins were transferred onto polyvinylidene difluoride membranes (no. IPVH00010; Millipore, Billerica, MA, USA) and developed by standard procedures. Levels of scanned films (no. 28906837; GE Healthcare, Buckinghamshire, UK) were adjusted in Adobe Photoshop CS4 Extended in accordance with the instructions given by this journal. P21 levels were analyzed densitometrically with Adobe Photoshop CS4 Extended using unmodified pictures of scanned films.

### Cell cycle analysis

Two-dimensional flow cytometry for BrdU and PI was performed as described previously.^23^

### Analysis of p21 expression during the cell cycle

Changes in p21 expression during different phases of the cell cycle were assessed by two-dimensional flow cytometry as follows: MCF7 cells were seeded in 6-well plates and treated as indicated. Afterwards, cells were collected following trypsinization (including floating cells) and spun down at 2500 r.p.m. for 5 min at room temperature (RT). Cell pellets were resuspended in PBS and crosslinked with formaldehyde. Cells were fixed for 10 min at 37 °C and subsequently chilled on ice for 1 min. To permeabilize cells, methanol was added to a final concentration of 90%. Cells were incubated for 30 min on ice and subsequently stored at −20 °C overnight. Later, cells were spun down at 2500 r.p.m. for 5 min at RT. Pellets were washed two times in incubation buffer (0.5% BSA in PBS) and spun down at 2500 r.p.m. for 5 min at RT. Samples were blocked in incubation buffer for 10 min at RT and an Alexa Fluor 488-conjugated p21 antibody or an Alexa Fluor 488-conjugated control rabbit IgG added. After incubating for 1 h at RT in the dark, incubation buffer was added and cells were spun down at 2500 r.p.m. for 5 min at RT. Cell pellets were resuspended in PBS containing 50 *μ*g/*μ*l RNase A and incubated for 1 h at RT in the dark. Finally, cells were stained with 25 μg/ml PI. Flow cytometry was performed using a Becton Dickinson FACScan and results analyzed using the BD CellQuest Pro software (San Jose, CA, USA).

### Clonogenic assays

For clonogenic assays, cells were seeded in 6-well plates and treated as indicated. Afterwards, cells were washed with growth medium and allowed to grow for 9 to 14 days. Subsequently, cells were fixed with methanol–acetone (1 : 1) at −20 °C for 10 min to overnight and stained with Giemsa solution for 5–8 min at RT. The area occupied by cell colonies was quantified by using the ImageJ software (National Institute of Mental Health, Bethesda, MD, USA). Brightness and contrast of scanned plates were adjusted in Adobe Photoshop CS4 Extended in accordance with the instructions given by this journal.

### Statistical analysis

Statistically significant differences between samples were determined by using an unpaired two-tailed Student's *t*-test.

## Abbreviations

*β*-gal: *β*-galactosidase
BrdU: 5-bromo-2′-deoxyuridine
BSA: bovine serum albumin
CPRG: chlorophenol red-*β*-𝒟-galactopyranoside
DMSO: dimethyl sulfoxide
EtOH: ethanol
GAPDH: glyceraldehyde 3-phosphate dehydrogenase
HDAC: histone deacetylase
HDM2: human MDM2
HNDFs: human normal dermal fibroblasts
HRP: horseradish peroxidase
iPS cell: induced pluripotent stem cell
KLF4: Krüppel-like factor 4
MDM2: murine double minute 2
OCT4: octamer-binding transcription factor 4
PI: propidium iodide
P/S: penicillin/streptomycin
RNase A: ribonuclease A
RT: room temperature
RT-PCR: reverse transcription-polymerase chain reaction
SAHA: suberoylanilide hydroxamic acid
SOX2: SRY (sex determining region Y)-box 2
TSA: trichostatin A
