# Supplementary material for: Incompatible effects of p53 and HDAC inhibition on p21 expression and cell cycle progression
Source: Cell Death Dis. 2013 Mar 7;4(3):e533–. doi: 10.1038/cddis.2013.61 (PMC3613839; doi:10.1038/cddis.2013.61)

Supplementary Figure S1

A

MCF7

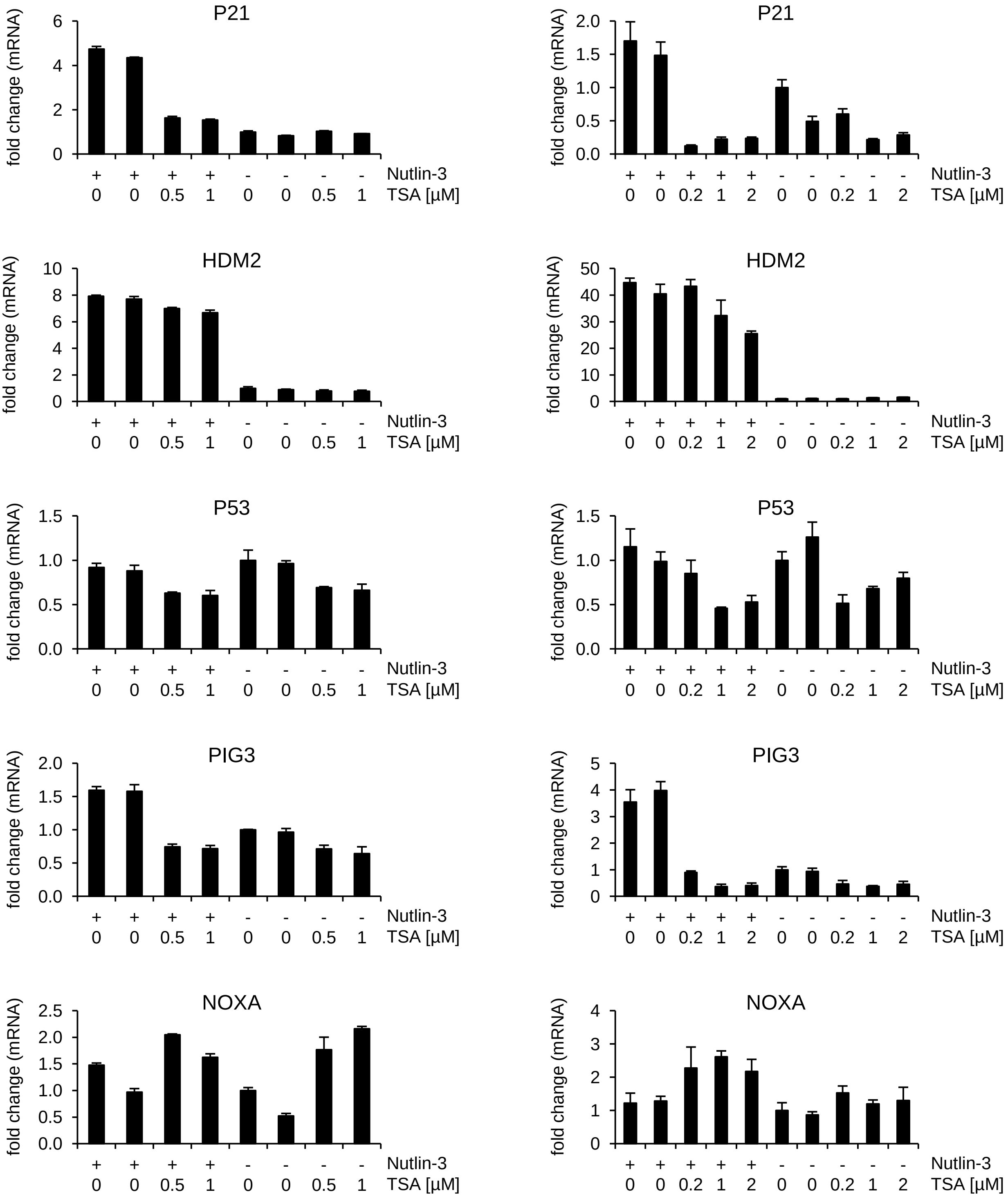

Supplementary Figure S1 (continued)

B

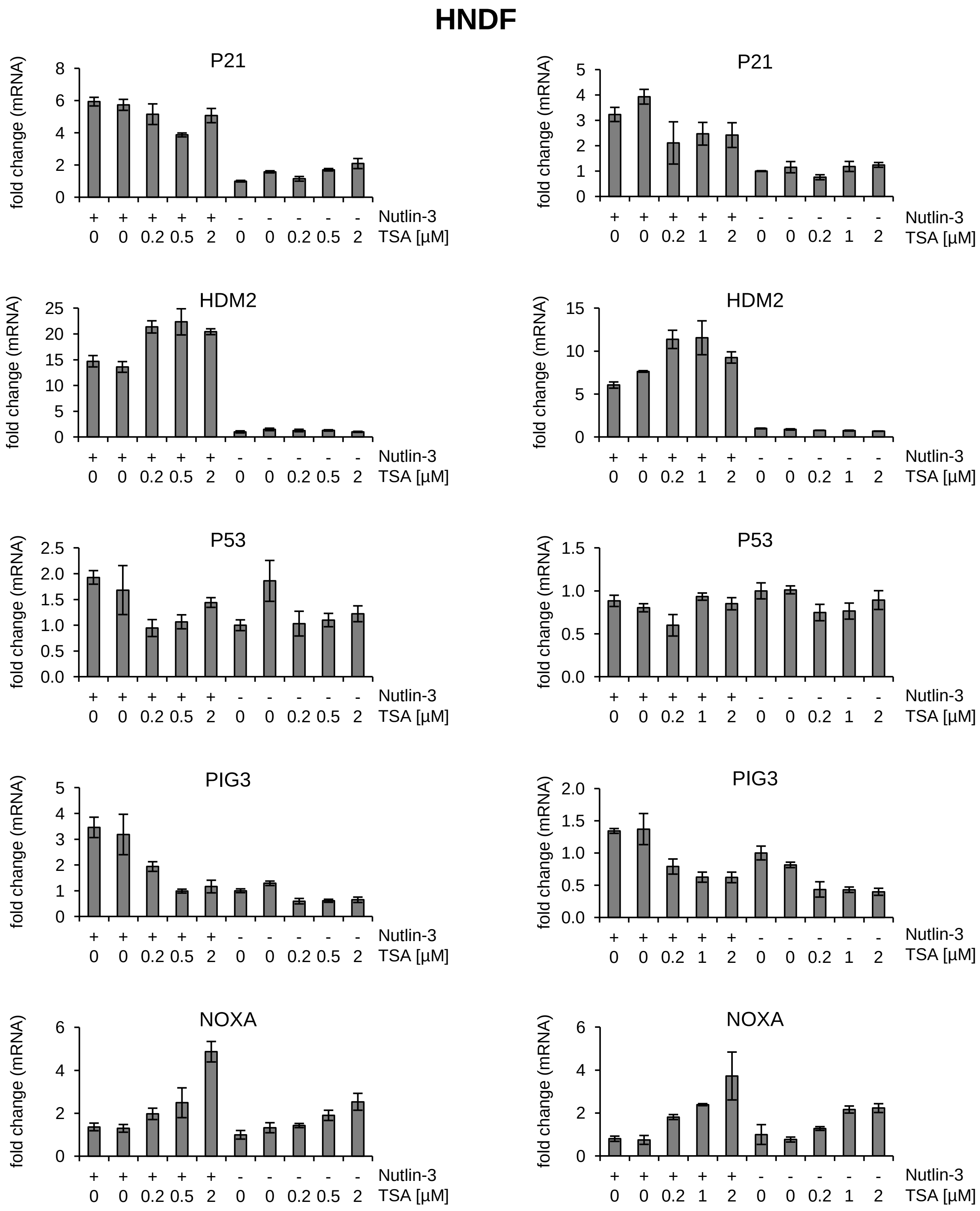

Supplementary Figure S1 (continued)

C **HCT116 p53$^{+/+}$**

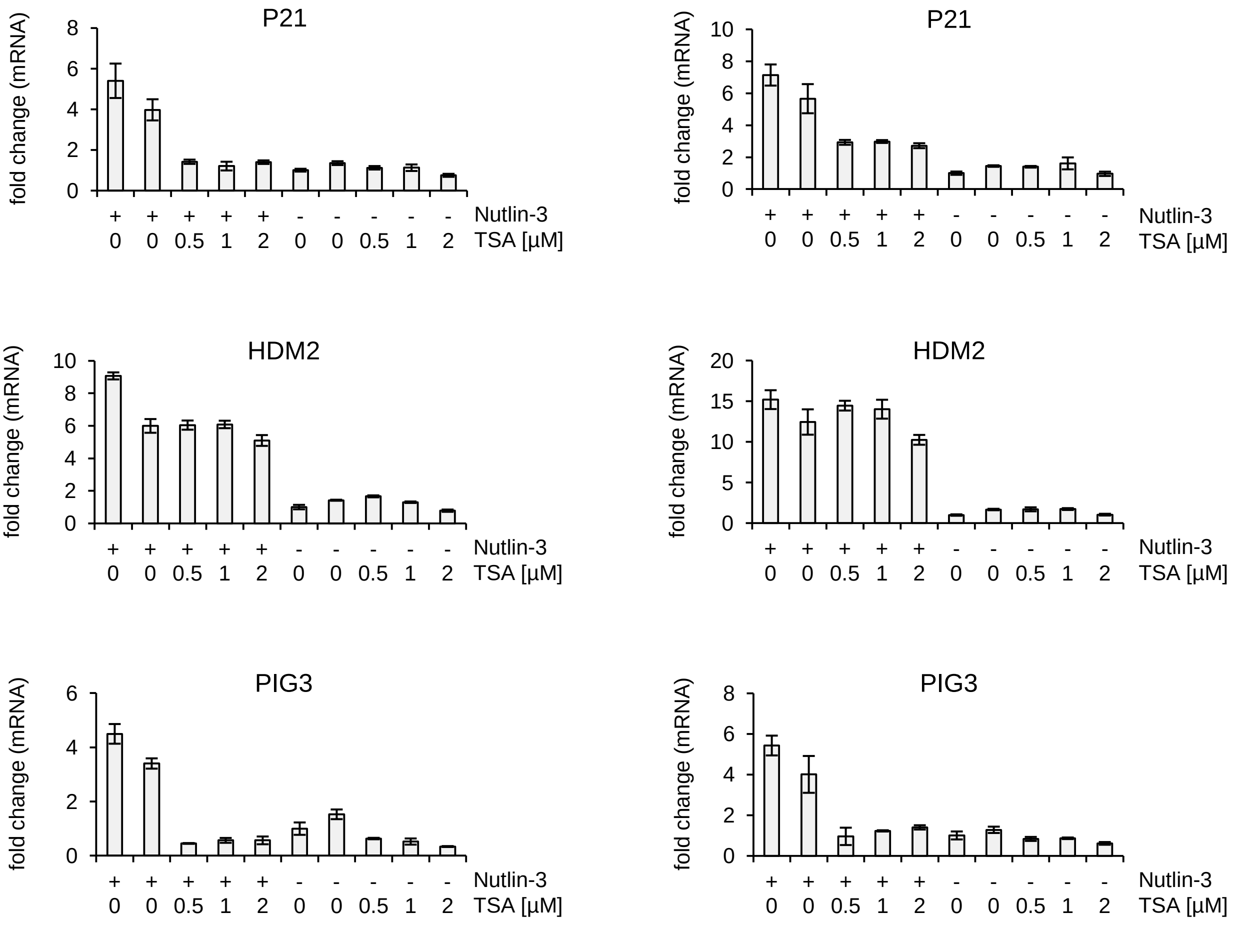

Supplementary Figure S1 (continued)

D

HCT116 p53$^{-/-}$

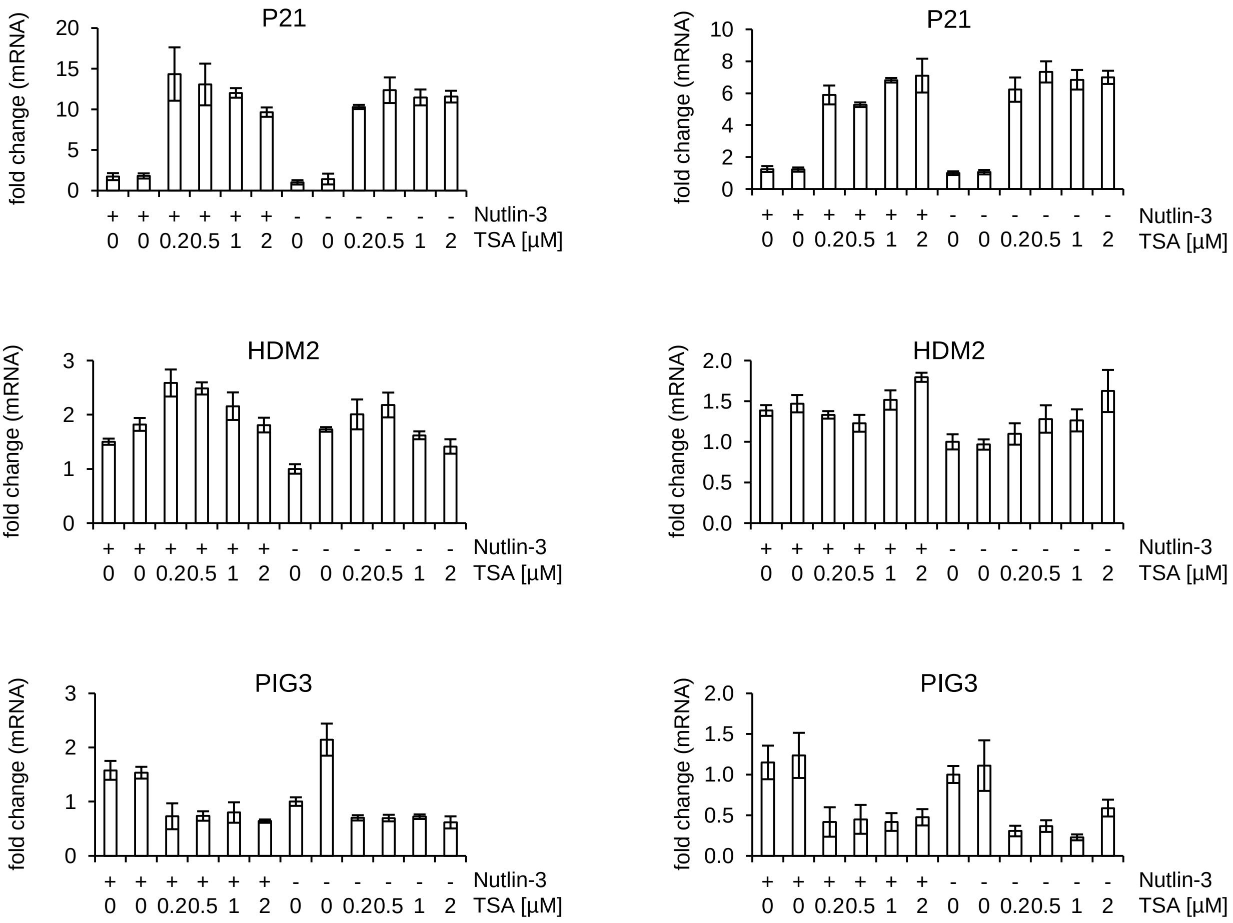

Supplementary Figure S2

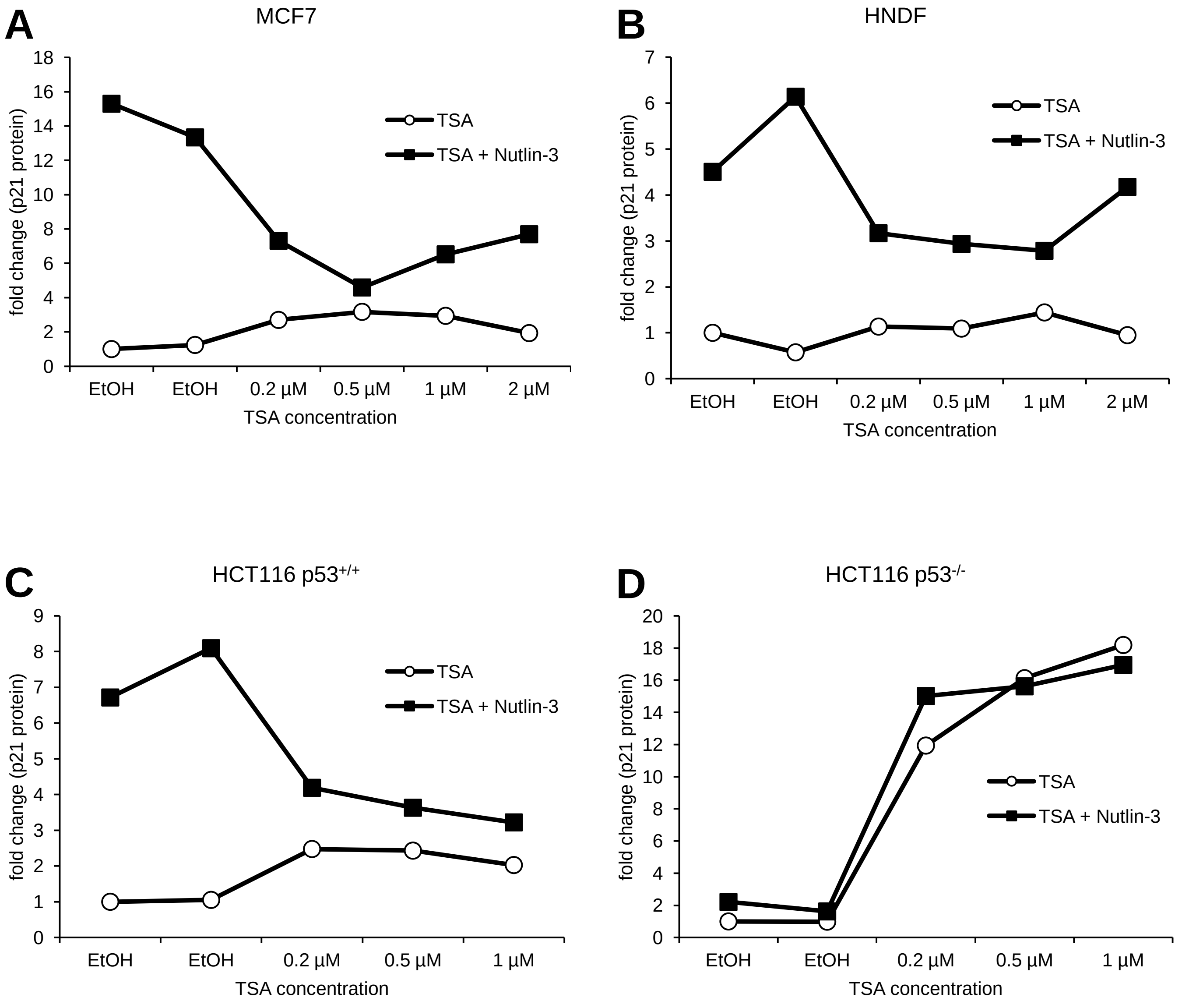

Supplementary Figure S3

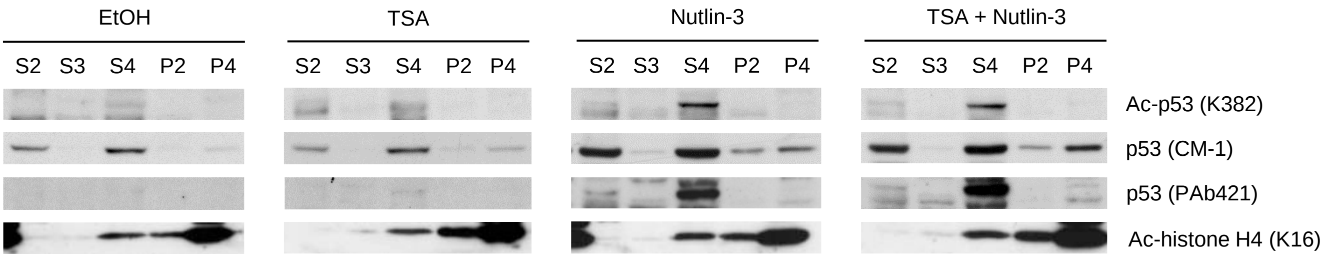

Supplementary Figure S4

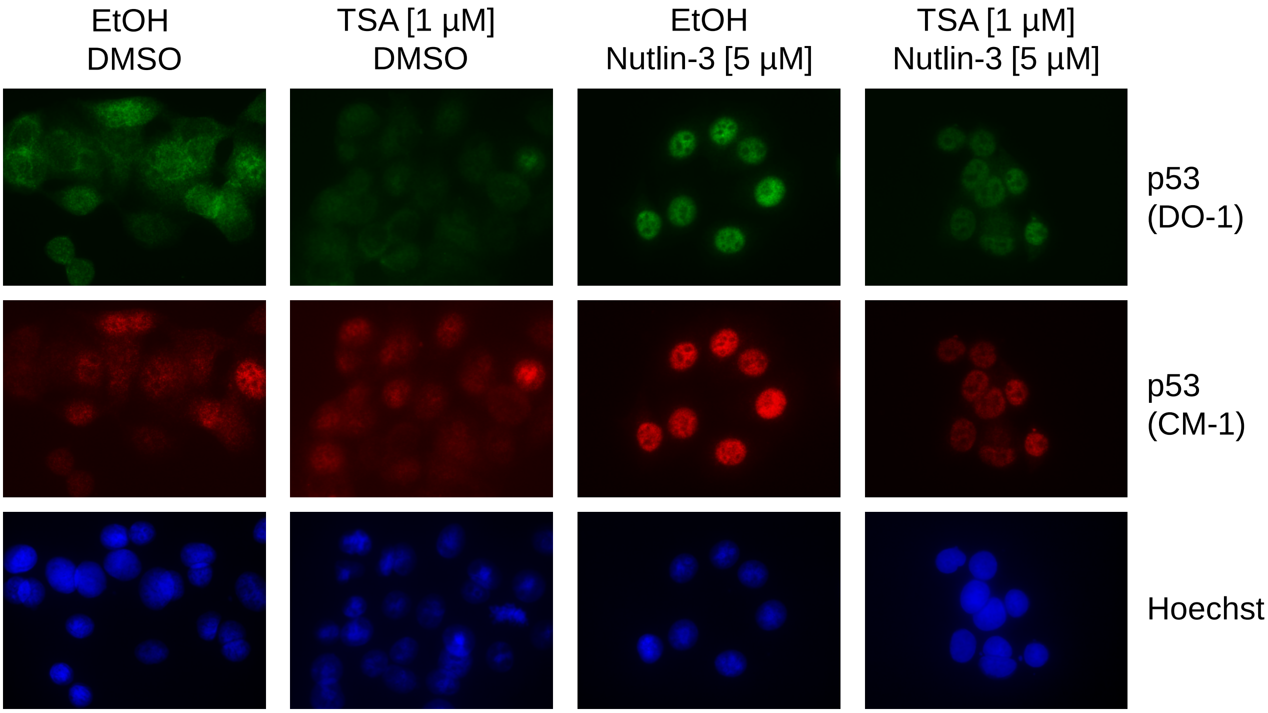

Supplementary Figure S5

A

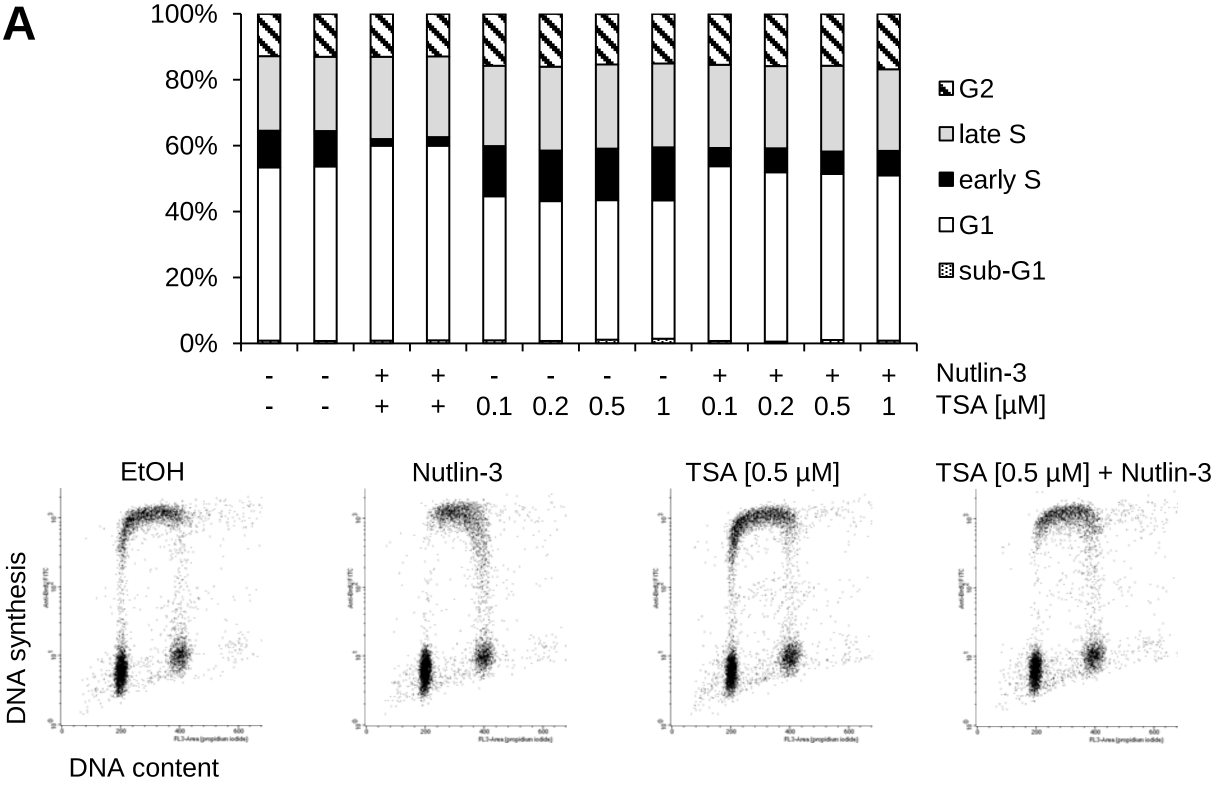

B

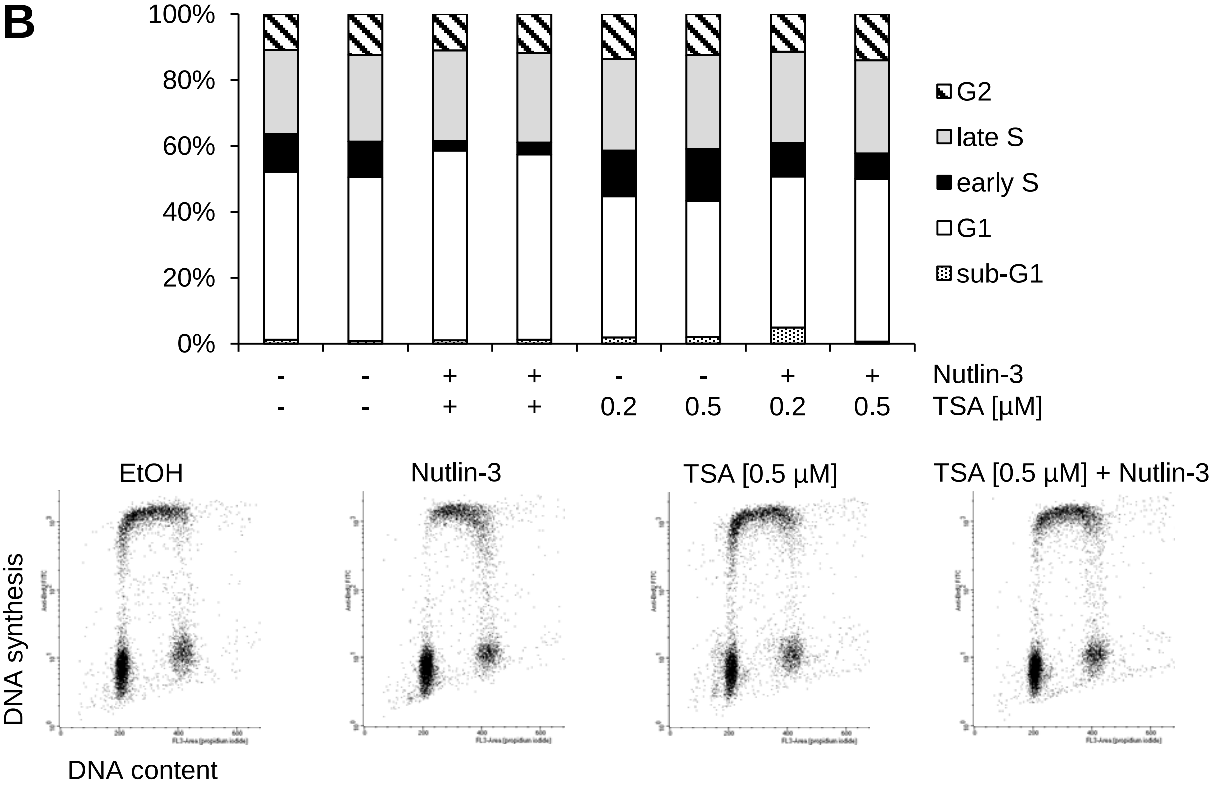

Supplementary Figure S6

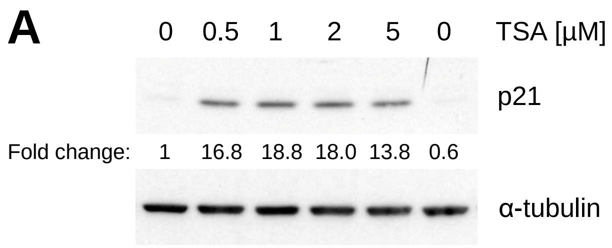

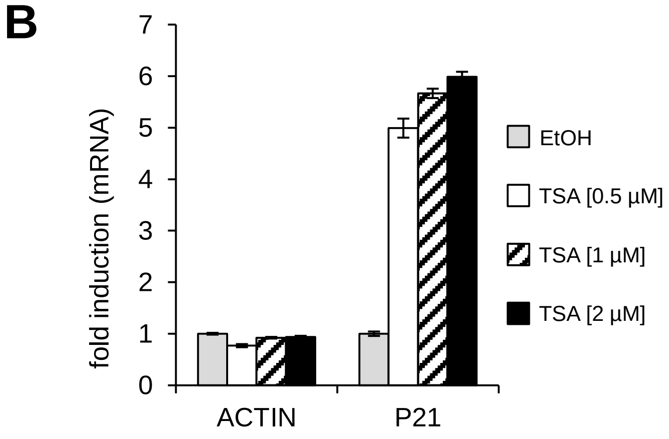

Supplementary Figure S7

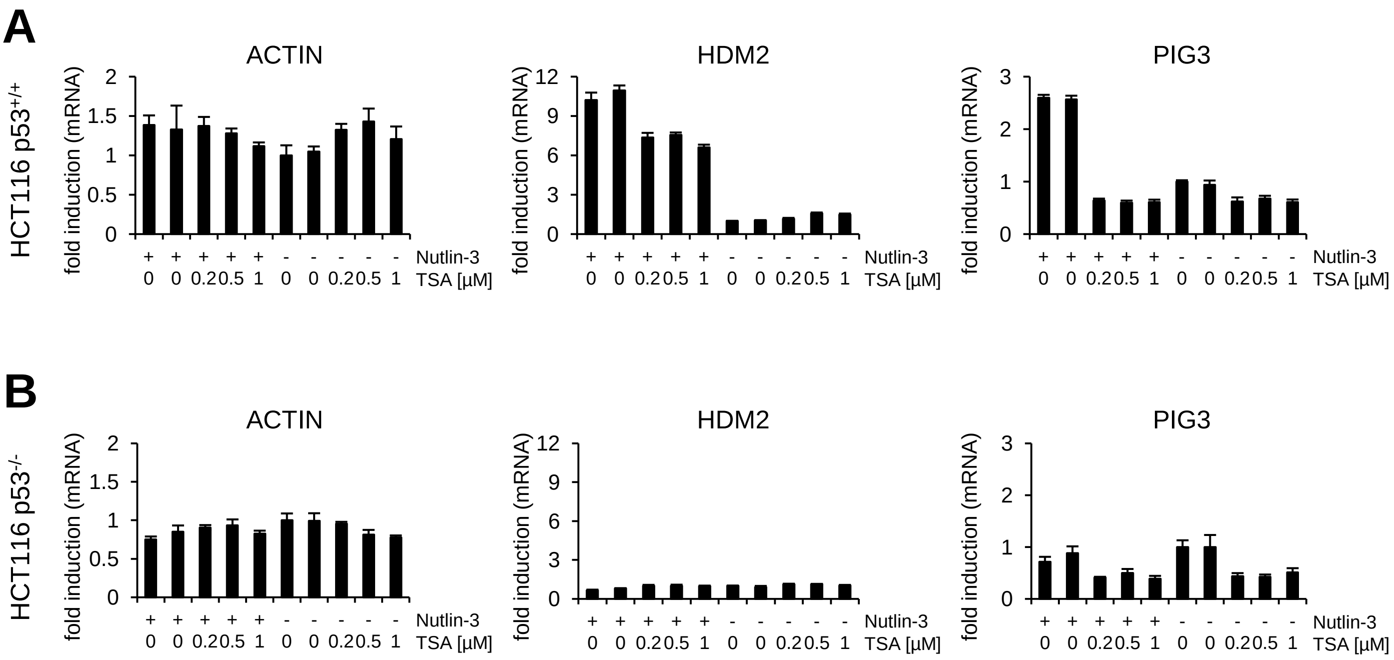

Supplement: Supplementary Figures [file cddis201361x1.pdf]
