# Supplementary material for: Incompatible effects of p53 and HDAC inhibition on p21 expression and cell cycle progression
Source: Cell Death Dis. 2013 Mar 7;4(3):e533–. doi: 10.1038/cddis.2013.61 (PMC3613839; doi:10.1038/cddis.2013.61)
Supplement: Supplementary Information [file cddis201361x2.pdf]

Supplementary Figure Legends

Supplementary Figure S1

Replicates of experiments presented in Figures 2, 3 and 5, respectively. (A) MCF7 cells, (B) HNDFs, (C) HCT116 p53$^{+/+}$ and (D) HCT116 p53$^{-/-}$ cells were treated as shown in Figure 2. mRNA levels were determined by quantitative RT-PCR as described in Materials and Methods.

Supplementary Figure S2

Densitometric analysis of p21 protein levels from Western blots presented in (A) Figure 2, (B) Figure 3, (C) Figure 5A and (D) Figure 5B, respectively. The p21 protein level of the first mock-treated sample of each experiment was set at 1.

Supplementary Figure S3

Various anti-p53 antibodies indicate that p53 protein levels and its subcellular localization do not change upon short-term treatment with TSA. MCF7 cells plated on 10 cm dishes were treated with TSA [0.1 µM] for 6 h; 1 h after TSA treatment, Nutlin-3 [5 µM] was added. Afterwards, cells were washed twice with PBS and scraped off the culture dish in PBS. Cells were spun down at 7000 rpm for 3 min and supernatants were discarded. Pellets were resuspended in 200 µl Buffer A (10 mM HEPES, pH 7.9; 10 mM KCl; 1.5 mM $MgCl_2$; 340 mM sucrose; 10% glycerol; 1 mM dithiothreitol (DTT); protease inhibitor cocktail (PIC, # 11836170001, Roche)) and incubated on ice for 5 min. Triton X-100 was added to a final concentration of 0.1%, followed by an incubation on ice for 8 min. Samples were centrifuged at 1300 x *g* at 4°C for 5 min. Supernatants (fraction S1) were further centrifuged at 20000 x *g* at 4°C for 5 min. The resulting supernatants (fraction S2) and pellets (fraction P2) were kept on ice until further processed. The pellets of the previous step (fraction P1) were washed once with 100 µl Buffer A (without DTT and PIC) and spun down at maximum velocity for 1 min. Supernatants were discarded and pellets lysed in 100 µl Buffer B (3 mM EDTA pH 8.0, 0.2 mM EGTA pH 8.0, 1 mM DTT and PIC) for 30 min on ice, followed by centrifugation at 1700 x *g* at 4°C for 5 min. Resulting supernatants (fraction S3) were kept on ice until further processed. Resulting pellets (fraction P3) was washed once with Buffer B (without DTT and PIC) and spun down at maximum velocity for 1 min. Supernatants were discarded and

pellets resuspended in 100 µl RIPA buffer (50 mM Tris/HCl, pH 7.5, 150 mM NaCl, 1% NP40, 0.1% sodium dodecyl sulfate (SDS), 0.5% deoxycholate and PIC), followed by incubation on ice for 10 min and subsequent centrifugation at maximum velocity for 10 min. Lithium dodecyl sulphate (LDS) containing 100 mM DTT was added to the resulting supernatants (fraction S4) and pellets (fraction P4) as well as all other fractions from previous steps. Afterwards, samples were heated at 70°C for 10 min and fractions P2 and P4 sonicated for 2 x 10 sec. Subsequently, Western blots were performed as described in the Materials and Methods section. To detect total p53 levels, antibodies PAb421 (1) and CM-1 (2) were used (both were a kind gift of David P. Lane). Antibody # 07-329 (Upstate, Lake Placid, NY, UK) was used to detect acetylated histone H4 (K16).

Supplementary Figure S4

The subcellular localization of p53 does not change upon short-term treatment with TSA. MCF7 cells seeded in 2-well glass object slides (Nunc) were treated as in shown in Figure 2A and stained as described previously (3). Pictures were taken with a Zeiss AxioVert 40C microscope equipped with a high-resolution AxioCam MRc5 camera. Brightness of images was adjusted with Adobe Photoshop CS4 Extended in accordance with instructions given by this journal.

Supplementary Figure S5

TSA weakens Nutlin-3 induced G1 arrest. MCF7 cells were either treated with vehicle (EtOH), Nutlin-3 [5 µM] for 6 h, TSA for 7 h, or with a combination of TSA and Nutlin-3 with Nutlin-3 being added 1 h after TSA for a total of 7 h. Samples were subjected to two-dimensional flow cytometric analysis following staining with BrdU and PI.

Supplementary Figure S6

TSA-induced p21 expression is independent of p53 status. MDA-MB-468 cells (p53R273H) were treated with TSA at various concentrations for 6 h. (A) p21 protein levels were determined by Western blotting, using α-tubulin (detected by antibody # T7451, Sigma-Aldrich) as loading control. Values below the p21 blot indicate changes in p21 protein levels as determined by densitometric analysis. (B) P21 mRNA levels were determined by quantitative RT-PCR with ACTIN as internal control. Error bars represent standard deviation.

Supplementary Figure S7

(A) HCT116 p53$^{+/+}$ and (B) HCT116 p53$^{-/-}$ cells were treated and analyzed as in Figure 2. mRNA levels were determined by quantitative RT-PCR. Error bars represent standard deviation.

References to Supplementary Figure Legends

1. Harlow E, Crawford LV, Pim DC, Williamson NM. Monoclonal antibodies specific for simian virus 40 tumor antigens. *J Virol* 1981 Sep; **39**(3)**:** 861-869.

2. Midgley CA, Fisher CJ, Bartek J, Vojtesek B, Lane D, Barnes DM. Analysis of p53 expression in human tumours: an antibody raised against human p53 expressed in Escherichia coli. *J Cell Sci* 1992 Jan; **101 (Pt 1):** 183-189.

3. Staples OD, Hollick JJ, Campbell J, Higgins M, McCarthy AR, Appleyard V*, et al.* Characterization, chemical optimization and anti-tumour activity of a tubulin poison identified by a p53-based phenotypic screen. *Cell Cycle* 2008 Nov 1; **7**(21)**:** 3417-3427.
